# Somatosensory perception in Visual Snow Syndrome: a comparison to age-, sex- and migraine-matched controls using Quantitative Sensory Testing - no evidence of altered somatosensory thresholds

**DOI:** 10.1186/s10194-025-02212-y

**Published:** 2025-11-06

**Authors:** Felix Fay, Ruth Ruscheweyh, Andreas Straube, Ozan Emre Eren

**Affiliations:** 1 Department of Neurology, LMU University Hospital, LMU Munich, Munich, Germany; 2 Department for Neurology, Bogenhausen Hospital, Munich, Germany

**Keywords:** Disturbed sensory filter, Tinnitus, Migraine, Visual snow, Somatosensory perception

## Abstract

**Background:**

Visual Snow Syndrome (VSS) is characterized by persistent, television noise like visual disturbances, accompanied by other visual and non-visual symptoms. Although VSS is recognized as a clinical entity, its underlying mechanisms remain poorly understood. One hypothesis is that VSS may involve increased sensory “noise” due to impaired sensory filtering, which could extend to non-visual modalities, such as somatosensory perception. This study aims to evaluate somatosensory perception in VSS using Quantitative Sensory Testing (QST), an established method to quantify sensory perception regarding different qualities such as heat, cold, touch and nociceptive stimuli.

**Methods:**

Thirty-six patients with VSS and thirty-nine controls matched for age, sex and migraine were included. Given the high prevalence of migraine in VSS patients, controls were matched for migraine for its potentially confounding effects in QST. We used the standard QST protocol to assess somatosensory perception, which we performed on the dorsum of the right hand. In addition to assessing sensory sensitivity, we also calculated intraindividual variability in test results (as a measure of “somatosensory noise”). For subgroup analysis both groups were divided by migraine status using a two-way ANOVA.

**Results:**

In demographics, migraine onset was earlier in VSS than in controls (11.7 years ± 6.5 vs. 17.8 years ± 9.1; *p* = 0.030), additionally migraine with visual aura was significantly more often present in VSS than in controls (80% vs. 23%; *p* = 0.017). Our two-way ANOVA analysis revealed no statistically significant main effect or interaction of migraine or VSS on QST parameters. Results for intraindividual variability were similar, except for a significant interaction between VSS and migraine on vibration detection threshold (F(1, 70) = 18.909, *p* < 0.001, partial η² = 0.21).

**Conclusions:**

These results suggest that VSS, despite being recognized as a multisensory disorder, seems to have no direct impact on somatosensory perception, supporting a predominantly higher-order condition of visual and sensory integration rather than a primary somatosensory dysfunction. Additionally, VSS patients showed significantly earlier migraine onset compared to controls, suggesting shared underlying mechanisms with migraine with aura, which would require further investigations.

**Supplementary Information:**

The online version contains supplementary material available at 10.1186/s10194-025-02212-y.

## Introduction

“Visual Snow” (VS) was first described 30 years ago. Patients reported countless tiny, flickering dots of varying colour and brightness within their visual field, which were described like the visual perception induced by a noisy TV screen. Initially falsely interpreted as a persistent variant of migraine aura [1], VS has become recognized as a distinct clinical entity, with a growing number of case descriptions [2]. Over time, a pattern of additional visual symptoms typically associated with VS emerged e.g. afterimages or trailing of moving objects (palinopsia), enhanced sensitivity to light (photophobia) or reduced vision at night (nyctalopia). This led to the first description of Visual Snow Syndrome (VSS) [3], quickly followed by its inclusion in the appendix of the third edition of the international classification of headache disorders (ICHD-3) (see Table 1**)** [4]. A number of further non-visual comorbidities have been described, including migraine (with or without aura), tinnitus, depression and anxiety disorder [5–7].

A key commonality among these symptoms is the increased sensory noise, particularly evident in Visual Snow and tinnitus, both of which can be conceptualized as a form of inappropriate sensory filtering. This hypothesis has been previously discussed by Morse et al. [8].

Given this, and the fact that disturbed sensory processing may extend to other sensory modalities, we investigated whether this “noisy” perception could also be detected in the processing of somatosensory information. Therefore, we used quantitative sensory testing (QST): a standardized protocol for assessing various sensory qualities through various thermal, epicritic and nociceptive stimuli [9, 10]. In this study, we used QST in VSS patients and migraine-matched controls to determine if and how VSS affects the perception and processing of sensory stimuli. Additionally, we investigated whether VSS patients exhibit greater variability in their responses to repeated somatosensory stimuli, as a sign of increased sensory noise or reduced filtering.

## Methods

### Participants

The study was conducted in accordance with the Declaration of Helsinki and was approved by the ethics committee of the medical faculty of Ludwig-Maximilians-University Munich (No. 22–0647). All patients gave their written in-formed consent prior to inclusion. Participants were recruited via VSS online self-help groups, from our VSS and headache outpatient clinic and by advertisements on the hospital campus and all subjects were clinically seen by an experienced neurologist.

Participants were divided into two groups consisting of VSS patients (*n* = 36) and controls (*n* = 39) that were matched for age, sex and migraine history. This matching aimed to control primarily for migraine as a potential confounder, given the high migraine comorbidity in VSS. For subgroup analyses, the participants were then later categorized by migraine status (with or without migraine).

Inclusion criteria were as follows: age between 18 and 50 years, and no major neurological or psychiatric conditions (apart from migraine or VSS) as determined by a structured clinical interview. We excluded participants over age 50 to reduce age-related variability in somatosensory perception and potentially confounding factors such as (sub)clinical neuropathy.

VSS and migraine (with or without aura) were diagnosed according to the ICHD-3 criteria [4] (see Table 1 for VSS). All migraineurs were assessed during the interictal period (headache-free for at least 48 h). Patients with a history of substance abuse were excluded to avoid the presence of Hallucinogen Persisting Perception Disorder (HPPD), which presents itself with symptoms similar to VSS [11]. On the day of testing, a total of seven VSS patients had taken medications, mainly for comorbidities (metoprolol, bisoprolol, fluoxetine, sertraline, duloxetine, aspirin, pantoprazole, budesonide and L-thyroxine), including two patients using lamotrigine as a trial for VSS. In contrast, no control participant had taken any medication on the day of the study.

### Study design

The study was conducted between October 2022 and November 2023. After the clinical interview, each participant completed a set of questionnaires, including the depression anxiety stress scales (DASS) [12], a quality of life measure (EQ-5D-5 L) [13], and, for those affected, migraine-related disability (MIDAS) [14]. Then, Quantitative Sensory Testing was performed. Detailed results from the here mentioned questionnaires have been reported in a prior study and are therefore not repeated here to avoid duplicate publication [15].

### Quantitative sensory testing (QST)

QST assessments were conducted following the protocol developed by the German Research Network on Neuropathic Pain (DFNS). Unless stated otherwise, we used the same equipment and followed the published protocol (Rolke et al. [9, 10]). Images of the respective tools can be found in [16]. All tests were performed at the dorsum of the right hand by the same investigator (FF). For each participant, the complete test serie was conducted twice to have sufficient data points to analyse the individual variability in sensory perception. Before each QST measurement, the upcoming test was explained in detail, and the measurement device was demonstrated to the participants. A 10-minute break between the two test series was observed to prevent habituation.

The protocol included a total of 7 tests, assessing 13 different parameters. The assessed parameters were cold and warm detection thresholds (CDT, WDT), the occurrence of paradoxical heat sensations (PHS) during alternating warm and cold stimuli (thermal sensory limen, TSL), cold and heat pain thresholds (CPT, HPT), mechanical detection thresholds (MDT), a stimulus-response curve for pinprick sensitivity (mechanical pain sensitivity, MPS) and dynamic mechanical allodynia (DMA), the wind-up ratio (WUR) reflecting pain amplification upon repetitive pinprick stimuli, vibration detection thresholds (VDT), and thresholds for pinprick (mechanical pain threshold, MPT) and blunt pressure (pressure pain threshold, PPT).

Thermal measurements were performed with a thermal sensory testing unit (TSA 2001-II MEDOC, Israel) with a 9.0 cm² contact thermode, ramped stimuli (1 °C/s), and cut-off temperatures of 0 °C and 50 °C. CDT, WDT, CPT and HPT were determined as the arithmetic mean of six measurements, with three repetitions performed in each of two test series. For TSL, the mean difference between cold and warm stimuli over both test series was calculated. MDT was assessed with a set of von Frey filaments (Optihair2-Set, Marstock Nervtest, Germany) ranging from 0.25 to 512 mN. The final threshold was set as the geometric mean of our total of ten series of ascending and descending stimuli across the two conducted test series.

The same calculus was performed for MPT, which we measured with a set of weighted pinprick stimuli ranging from 8 to 512 mN. MPS was determined using a randomized sequence of pinprick stimuli, with three non-painful stimuli (namely a cotton wisp, a cotton wool tip, and a brush) interspersed to assess ALL. Participants rated pain responses on a 0-100 numerical rating scale after each stimulus. WUR was calculated by comparing pain ratings between a single 256mN stimulus and a series of 10 repeated 256mN stimuli applied at 1 Hz (ratio single / mean of series of ten). VDT was assessed using a neurological tuning fork applied to the styloid process of the radius and determined as the mean of our 10 measurements across the 2 test series, with each measurement recording the point where vibration was no longer felt.

PPT was assessed with a hand-held pressure algometer (FDN200, Wagner Instruments, USA) with a surface area of 1 cm^2^. The pressure was gradually applied to the thenar muscle on the palm until the participant reported the first sensation of pain. This was repeated a total of 3 times in each test serie, the final PPT value was calculated as the mean of these trials.

### Statistics and further processing

Statistics were conducted in Excel and Jamovi (Version 2.23.8) [17] and graphics were prepared using PyCharm (Version 2024.1.1) [18].

Logarithmic transformations were performed for CDT, WDT, TSL, MDT, MPT, MPS, WUR, and PPT values as required by the QST analysis protocol [9]. Two-way ANOVA was performed with factors group (VSS vs. controls) and migraine status (yes + vs. no -) for each of the 11 QST parameters, followed by Bonferroni correction for 11 tests. Because of the different prevalence of migraine with aura between groups, aura status (yes/no) was included as an additional cofactor within the ANOVA. Adjusting for aura did not change the findings.

For group comparisons of demographic data and after assessing the distribution of continuous variables with the Shapiro-Wilk Test, Mann-Whitney-U test (MWU) was used for continuous variables, while Chi-Square Test was applied to categorical variables in Table 2. For visualization of QST data, we Z-transformed the average result of each test for each participant of the VSS+/Migraine+, VSS+/Migraine-, VSS-/Migraine + groups with respect to the VSS-/Migraine- group and plotted average Z-scores. In both instances, we inverted the sign where necessary, so that values above the reference line indicate increased sensitivity, while negative values reflect reduced sensitivity to the respective stimuli, a common practice in QST literature [9]. Finally, we investigated intraindividual variability between measurements by calculating the variance of each test result within each participant and comparing the average variance of each test between groups using a two-way ANOVA with factors VSS and migraine as above. No variance could be calculated for the WUR, where the QST protocol provides only a single value per participant.

## Results

**Fig. 1:**
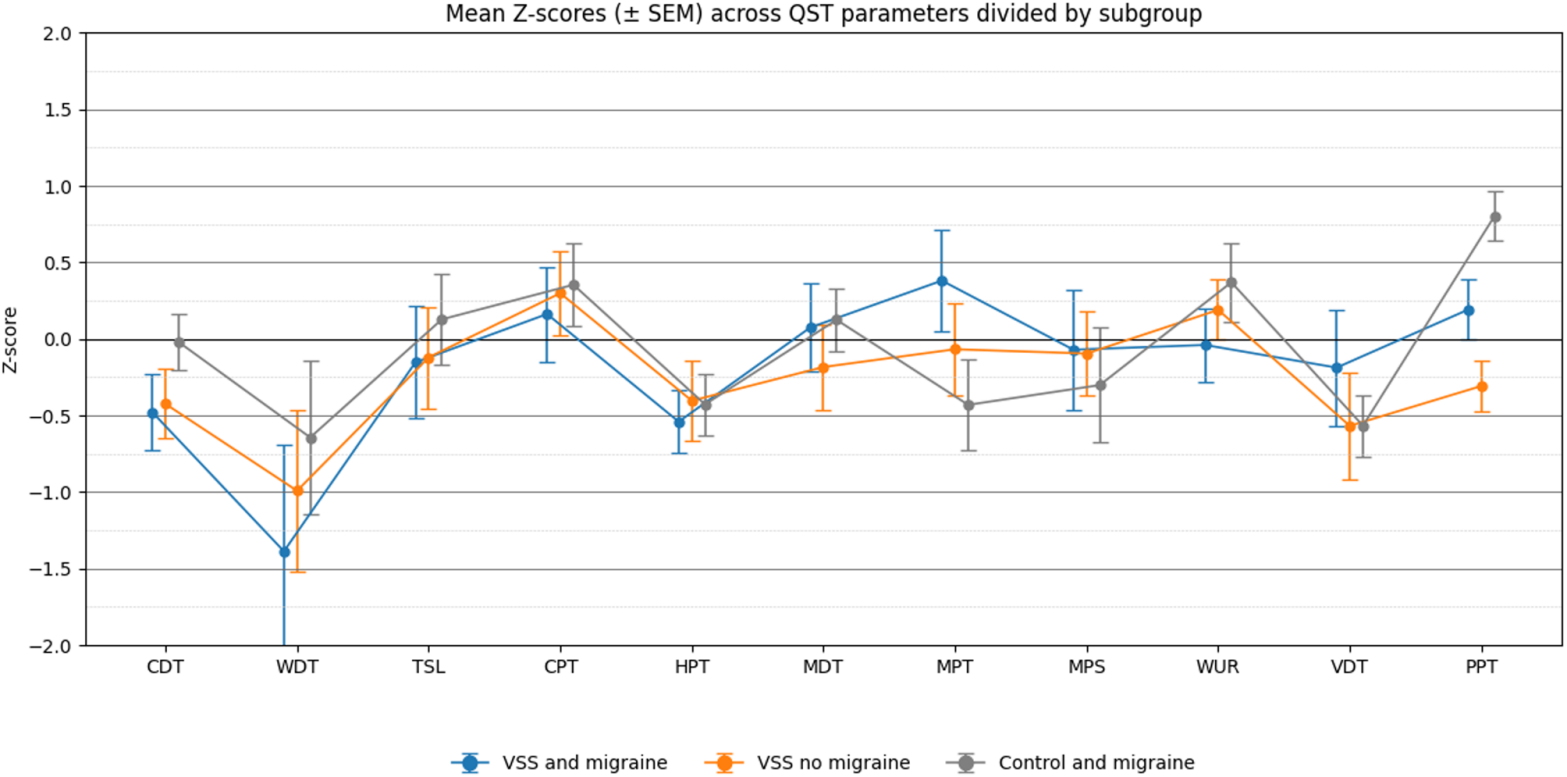
Sensory profiles presented as mean Z-scores ± standard error of mean in different subgroups. Z-scores reflect the relative sensory sensitivity of subgroups patients compared to controls with no migraine. Values above the reference line represent increased sensitivity, while values below indicate decreased sensory sensitivity. Z-scores were inverted where necessary so that Z-scores above 0 indicate an increased sensitivity

Mean and standard deviations of QST results within groups are shown in Table 3. Neither DMA nor PHS were observed in any participant and thus not further analysed. Z-Scores are illustrated Fig. 1. Numerical Z-Scores results can be found in supplementary material Table 5. Intraindividual variance for each QST parameter including mean values, confidence intervals and p-values are shown in Table 4.

### Demographics

While age, sex and migraine distribution were similar in both groups, there was a significant difference in the age at first manifestation of migraine (VSS patients 11.7 ± 6.5 years vs. Controls 17.8 ± 9.1 years; *p* = 0.03). Additionally, among those with migraine history, migraine with aura was significantly more frequent in VSS patients compared to controls (80% vs. 25%; *p* = 0.017). Detailed information on clinical features of VSS and migraine can be found in Table 2.

### Sensory detection thresholds

VSS patients showed no major abnormalities in sensory detection thresholds compared to controls. CDT was slightly lower (~ 0.4–0.5 °C) in patients with VSS. For WDT, both VSS subgroups showed a similar trend (~ 0.7–1.2 °C higher). MDT, WUR and VDT showed almost identical measurements across all subgroups.

Apart from an effect of VSS on CDT, which did not hold after correction for multiple testing (*p* = 0.057 uncorrected, *p* = 0.627 after Bonferroni adjustment), the two-way ANOVA revealed no significant main or interaction effects of either VSS or migraine on our sensory detection parameters.

### Pain thresholds and sensitivity

VSS patients showed no major abnormalities in pain thresholds compared to controls and across subgroups. For cold (CPT) and heat pain (HPT) all subgroups clustered around ~ 22 °C and ~ 45 °C, respectively, with ≤ 2 °C spread. MPT and MPS showed almost identical results across subgroups, with only minor variations. Interestingly the mean values for PPT showed strong variations with differences of up to 20 N/cm² between subgroups. However, these observed differences did not survive correction for multiple testing. In total, no main or interaction effects for PPT remained after Bonferroni correction (Table 3).

### Intraindividual variance

To explore the possibility of increased “sensory noise”, we calculated the intra-individual variance for every QST parameter and performed 2 × 2 ANOVA analysis. Out of all parameters, before correction, the interaction between VSS and migraine reached nominal significance for CDT, PPT and VDT variability (all uncorrected *p* < 0.05). After correction, however, only the VDT interaction remained significant (*p* < 0.001) as shown in Table 4. Interestingly, migraine increased the variability in controls but had the opposite effect in VSS.

## Discussion

Despite the characteristic sensory disturbances of VSS, no objective neurological deficits have been consistently demonstrated [2, 5, 19]. Ophthalmological and neuroimaging findings are typically normal [20, 21]. Consistent with this, our QST study showed no significant differences in somatosensory perception between VSS and migraine matched controls.

Additionally, we investigated intraindividual variability as a potential indicator of “sensory noise”. Except for a significant interaction of VSS and migraine in the variability of Vibration Detection Threshold (VDT), which likely reflects an artefact due to the small subgroup size, there were no significant effects. Overall, these findings indicate that primary somatosensory processing remains intact in VSS.

Interestingly, previous imaging studies revealed altered activity and connectivity in several key regions involved in sensory processing and integration including the visual association and motion networks, thalamo-cortical pathways and core parts of the salience networks, such as the insula and the posterior cingulate cortex, but also the precuneus [21–24]. That supports the concept that VSS involves a sensory dysregulation of higher order and not a basic somatosensory deficit. There are parallels to tinnitus, where in many cases there is perception of persistent “internal noise” despite normal peripheral findings [8, 25]. Notably, signs of activation have also been reported in the left primary auditory cortex and other areas involved in the development and sustaining to tinnitus, suggesting overlapping mechanisms [26–28]. Similarly, VSS may reflect abnormal filtering or integration within visual and associated cortical networks, despite the absence of detectable changes in somatosensory testing.

A secondary observation was the younger age of migraine onset among VSS patients, consistent with previous studies reporting earlier onset in migraine with aura [29, 30]. This is particularly relevant, as up to 72% of VSS patients experience migraine, with more than half of them reporting migraine with aura [31]. In our VSS group, visual aura was significantly more frequent, supporting these findings of earlier migraine onset in aura patients. This may indicate shared modulatory mechanisms, such as the cortical hyperexcitability discussed above. However, this remains speculative and should be systematically explored in aura-matched controls.

To our knowledge, this is the first study to report a significant difference in migraine onset age within a well-characterized VSS cohort.

### Limitations

There were some limitations to our study. The recruitment was based on self-help groups on the internet and patients from our specialized visual snow clinic. This may have led to the inclusion of a more severely affected patient cohort. Although the sample size in our study was comparable to or even slightly larger than many previous studies on VSS, we believe these findings should still be replicated in a larger cohort as smaller differences between groups may have remained undetected, particularly in subgroup analyses where sample sizes were further reduced. Another potential limitation is the use of medications in some VSS participants, primarily for comorbid conditions, although there is no substantial evidence that any of these medications interfere with QST results and indeed we did not find significant differences. Regarding demographics, the control group included individuals both with and without migraine to match the comorbidity profile of the VSS group. While using only healthy controls might have provided a cleaner comparison, we matched for migraine to control for its potential confounding effects, given its high prevalence in VSS. We believe this approach offers a more clinically relevant comparison. Unfortunately matching for aura was not performed, resulting in significantly higher portion of aura in VSS compared to controls. While we accounted for this by including aura status as a covariate in our ANOVA, the small number of aura patients in the control group did not allow for a meaningful subgroup comparison in the analysis of our results. Future work should recruit larger aura-balanced samples and focus on objective aura documentation to determine commonalities and differences in VSS and migraine aura to determine their relation and interactions. Additionally, sex-related differences in somatosensory processing could represent a potential confounder, however, the higher proportion of female participants in the control group did not reach statistical significance.

## Conclusion

Our findings demonstrate normal somatosensory thresholds and variability in Visual Snow Syndrome supporting the current understanding of VSS being a predominantly disorder of visual and sensory integration without substantial involvement of the primary somatosensory system. The analogy to tinnitus as “perception of noise” without peripheral objectifiable dysfunction, highlights a higher order origin.

As a side finding, we observed significantly earlier migraine onset in VSS patients compared to controls. This aligns with prior reports on migraine with aura and may indicate shared mechanisms, such as cortical hyperexcitability or a confounding effect due to high aura prevalence, needing further investigation.

**Table 1:** Criteria for the definition of the visual snow syndrome, according to ICHD-3 (diagnosis A1.4.6)

A	**Visual snow**: dynamic, continuous, tiny dots in the entire visual field lasting longer than 3 months
**B**	Presence of at least two additional visual symptoms of the four following categories:
	i. **Palinopsia**: At least one of the following: afterimages or trailing of moving objects.
	ii. **Enhanced entoptic phenomena. At least one of the following**: Excessive floaters in both eyes, excessive blue field entoptic phenomenon, self-light of the eye, or spontaneous photopsia.
	iii. **Photophobia**: enhanced sensitivity to light
	iv. **Nyctalopia**: impaired or reduced vision at night
**C**	Symptoms are not consistent with typical migraine visual aura.
**D**	Symptoms are not better explained by another disorder.

**Table 2:** Clinical characteristics of VSS patients and controls

	VSS patients		Controls		*p*
Migraine	+	-	+	-	
Participants	*n* = 15	*n* = 21	*n* = 16	*n* = 23	
**Demographic data**					
Female (%)	8 (53%)	8 (38%)	12 (75%)	13 (56%)	0.053
Age in years (mean ± SD)	31.2 ± 7.5	32.1 ± 8.1	35.6 ± 9.0	33.9 ± 8.6	0.190
Migraine Prevalence (%)	15 (42%)		17 (43%)		0.955
**Migraine Characteristics**					
Migraine with Aura (%)	12 (80%)	-	4 (23%)	-	0.017
- Visual Aura (%)	12 (100%)	-	4 (100%)	-	-
- Other Aura	-	-	-	-	
Age at Migraine Onset (years, mean ± SD)	11.7 ± 6.5	-	17.8 ± 9.1	-	0.030
Migraine Frequency (attacks/month)	9.02 ± 8.2	-	6.86 ± 5.3	-	0.900
Average Headache Intensity	5.75 ± 2.0	-	6.64 ± 1.5	-	0.576
**Clinical Features of VSS**					
Age at VSS Onset (years, mean ± SD)	20.1 ± 8.9	26.35 ± 9.4	-		0.811
Palinopsia (%)	12 (80%)	18 (85.7%)	-		0.230
Nyctalopia (%)	10 (66.7%)	7 (33.3%)	-		0.101
Entoptic Phenomena (%)	9 (60%)	6 (28.6%)	-		0.332
Photophobia (%)	14 (93.3%)	17 (81.0%)	-		0.829
Light flashes	5 (33.3%)	3 (14.3%)	-		0.244
Halos	1 (6.7%)	2 (9.5%)	-		0.686
Derealisation	-	1 (4.8%)	-		0.364
Tinnitus (%)	11 (73.3%)	17 (81%)	-		0.244

Statistical comparisons were performed using the Mann-Whitney U-Test for continuous variables and the Chi-Square Test for categorical variables. P-values refer to comparisons between the overall VSS cohort and the overall control group, except for the category “Clinical features of VSS” where they compare the subgroups VSS migraine and VSS no migraine. All percentage values presented (e.g., for migraine prevalence, gender etc…) refer to the respective subgroup and not to the total study population

**Table 3:** Mean values and standard deviation of QST parameters stratified by Visual Snow, migraine status and corresponding two-way ANOVA p-values for main effects and their interaction

VSS	Migraine	CDT	WDT	TSL	CPT	HPT	MDT	MPT	MPS	WUR	VDT	PPT
+	+	-2.13 ± 1.02	3.49 ± 2.64	4.93 ± 3.01	21.05 ± 7.73	45.33 ± 2.35	0.47 ± 0.34	209.51 ± 94.80	3.12 ± 2.50	2.43 ± 1.09	7.44 ± 0.56	63.03 ± 14.46
	-	-2.07 ± 1.11	3.10 ± 2.34	5.17 ± 2.91	21.92 ± 8.04	44.93 ± 3.54	0.53 ± 0.35	261.46 ± 126.99	3.17 ± 4.38	2.66 ± 1.00	7.30 ± 0.60	73.17 ± 17.79
-	+	-1.64 ± 0.81	2.77 ± 1.94	4.78 ± 3.06	22.28 ± 7.00	45.00 ± 2.33	0.42 ± 0.17	298.24 ± 136.15	2.92 ± 3.12	2.96 ± 1.41	7.30 ± 0.30	52.38 ± 10.36
	-	-1.63 ± 1.06	2.34 ± 1.35	3.85 ± 1.79	19.98 ± 6.63	43.87 ± 3.04	0.49 ± 0.30	246.10 ± 111.64	2.74 ± 2.20	2.50 ± 1.08	7.51 ± 0.39	68.51 ± 20.41
**Two-Way ANOVA: Effect of Visual Snow**,** Migraine**,** and Their Interaction (Bonferroni-corrected p-values for 11 comparisons)**												
Effect of VSS		0.627	1.000	1.000	1.000	1.000	1.000	1.000	1.000	1.000	1.000	0.264
Effect of Migraine		1.000	1.000	1.000	1.000	1.000	1.000	1.000	1.000	1.000	1.000	0.088
Effects of VSS x Migraine		1.000	1.000	1.000	1.000	1.000	1.000	1.000	1.000	1.000	1.000	1.000

CDT and WDT are measured in °C relative to baseline; TSL, CPT, and HPT in absolute °C; MDT and MPT in mN; MPS as a score out of 100; WUR is unitless; VDT as a score out of 8; and PPT in N/cm². For ANOVA Analysis, all variables were log transformed, except CPT, HPT and VDT, as described by Rolke et al. [10]. Additionally, CDT and WDT values were multiplied by -1 prior to log transformation so that higher numbers represent higher temperatures thresholds and are reported with positive numbers in the manuscript

**Table 4:** Mean individual variance and standard deviation of QST parameters stratified by Visual Snow, migraine status and corresponding two-way ANOVA p-values for main effects and their interaction

VSS	Migraine	CDT	WDT		TSL		CPT		HPT		MDT		MPT			MPS		WUR		VDT		PPT
+	+	0.85 ± 0.90	1.03 ± 2.05		15.14 ± 14.93		10.34 ± 29.06		1.40 ± 1.86		1.00 ± 2.63		18748.18 ± 5638.78			91.38 ± 131.34		2.31 ± 5.34		0.14 ± 0.14		31.13 ± 36.70
	-	0.86 ± 1.17	1.47 ± 3.11		24.48 ± 35.11		2.67 ± 1.97		1.34 ± 2.00		1.04 ± 2.07		19203.98 ± 7586.03			81.83 ± 201.59		8.21 ± 22.07		0.24 ± 0.08		64.50 ± 71.00
-	+	0.69 ± 1.14	0.79 ± 1.60		17.69 ± 19.14		3.32 ± 2.97		1.92 ± 4.68		0.46 ± 1.54		14060.80 ± 6496.81			103.39 ± 148.73		9.39 ± 11.19		0.35 ± 0.20		77.36 ± 51.71
	-	0.54 ± 0.78	0.37 ± 0.59		20.30 ± 24.95		10.60 ± 34.88		1.14 ± 1.41		0.49 ± 0.95		14946.97 ± 9414.52			66.26 ± 107.61		10.44 ± 22.84		0.16 ± 0.14		39.46 ± 36.37
**Two-Way ANOVA: Effect of Visual Snow**,** Migraine**,** and Their Interaction (Bonferroni-corrected p-values for 11 comparisons)**																						
Effect of VSS		1.000		1.000		1.000		1.000		1.000		1.000		1.000	1.000		1.000		0.429		1.000	
Effect of Migraine		1.000		1.000		1.000		1.000		1.000		1.000		1.000	1.000		1.000		1.000		1.000	
Effects of VSS x Migraine		1.000		1.000		1.000		1.000		1.000		1.000		1.000	1.000		1.000		< 0.001		0.055	

## Electronic Supplementary Material

Below is the link to the electronic supplementary material.

Supplementary Material 1

## Data Availability

The datasets used during the current study are available from the corresponding author upon reasonable request. Data can only be shared in a pre-processed and anonymised format.

## Abbreviations

CDT: Cold Detection Threshold
CPT: Cold Pain Threshold
DMA: Dynamic Mechanical Allodynia
HPT: Heat Cold Pain Threshold
MDT: Mechanical Detection Threshold
MPS: Mechanical Pain Sensitivity
MPT: Mechanical Pain Threshold
MWU: Mann-Whitney-U Test
PHS: Paradoxical Heat Sensation
PPT: Pressure Pain Threshold
QST: Quantitative Sensory Testing
TSL: Temperature Sensory Line
VDT: Vibration Detection Threshold
VEP: Visual Evoked Potential
VSS: Visual Snow Syndrome
WDT: Warm Detection Threshold
WUR: Wind-Up Ratio

## References

[1] Liu GT, Schatz NJ, Galetta SL et al (1995) Persistent positive visual phenomena in migraine. Neurology 45:664–668. 10.1212/WNL.45.4.664 7723952 10.1212/wnl.45.4.664

[2] Schankin CJ, Maniyar FH, Digre KB, Goadsby PJ (2014) Visual snow’ – a disorder distinct from persistent migraine aura. Brain 137:1419–1428. 10.1093/brain/awu050 24645145 10.1093/brain/awu050

[3] Schankin CJ, Maniyar FH, Sprenger T et al (2014) The relation between migraine, typical migraine aura and visual snow. Headache 54:957–966. 10.1111/head.12378 24816400 10.1111/head.12378

[4] (2018) Headache Classification Committee of the International Headache Society (IHS) The International Classification of Headache Disorders, 3rd edition. Cephalalgia 38:1–211. 10.1177/0333102417738202 10.1177/0333102417738202 29368949

[5] Puledda F, Schankin C, Goadsby PJ (2020) Visual snow syndrome: A clinical and phenotypical description of 1,100 cases. Neurology 94:e564–e574. 10.1212/WNL.0000000000008909 31941797 10.1212/WNL.0000000000008909 PMC7136068

[6] Klein A, Schankin CJ (2021) Visual snow syndrome as a network disorder: A systematic review. Front Neurol 12:724072. 10.3389/fneur.2021.724072 34671311 10.3389/fneur.2021.724072 PMC8521005

[7] Solly EJ, Clough M, Foletta P et al (2021) The psychiatric symptomology of visual snow syndrome. Front Neurol 12 10.3389/fneur.2021.703006 PMC8362098 34393980

[8] Morse K, Vander Werff KR (2024) Cortical auditory evoked potential indices of impaired sensory gating in people with chronic tinnitus. Ear Hear 45:730. 10.1097/AUD.0000000000001463 38273451 10.1097/AUD.0000000000001463

[9] Rolke R, Magerl W, Campbell KA et al (2006) Quantitative sensory testing: a comprehensive protocol for clinical trials. Eur J Pain 10:77–77. 10.1016/j.ejpain.2005.02.003 16291301 10.1016/j.ejpain.2005.02.003

[10] Rolke R, Baron R, Maier C et al (2006) Quantitative sensory testing in the German research network on neuropathic pain (DFNS): standardized protocol and reference values. Pain 123:231–243. 10.1016/j.pain.2006.01.041 16697110 10.1016/j.pain.2006.01.041

[11] Ford H, Fraser CL, Solly E et al (2022) Hallucinogenic persisting perception disorder: A case series and review of the literature. Front Neurol 13:878609. 10.3389/fneur.2022.878609 35599738 10.3389/fneur.2022.878609 PMC9120359

[12] Lovibond SH (1995) Manual for the depression anxiety stress scales. Sydney Psychology Foundation

[13] Szende A, Janssen B, Cabases J (2014) Self-Reported population health: an international perspective based on EQ-5D. Springer, Dordrecht (NL) 29787044

[14] Stewart WF, Lipton RB, Dowson AJ, Sawyer J (2001) Development and testing of the migraine disability assessment (MIDAS) questionnaire to assess headache-related disability. Neurology 56:S20–28. 10.1212/wnl.56.suppl_1.s20 11294956 10.1212/wnl.56.suppl_1.s20

[15] Fay F, Straube A, Ruscheweyh R, Eren OE (2024) [Characterization of a German cohort with visual snow syndrome]. Nervenarzt 95:1124–1130. 10.1007/s00115-024-01768-5 39532711 10.1007/s00115-024-01768-5

[16] Mücke M, Cuhls H, Radbruch L et al (2021) Quantitative sensory testing (QST). Engl Version Schmerz 35:153–160. 10.1007/s00482-015-0093-2 10.1007/s00482-015-0093-2 26826097

[17] The jamovi project (2024) jamovi (Version 2.23) [Computer Software]. Retrieved from https://www.jamovi.org

[18] PyCharm by JetBrains Available from: https://www.jetbrains.com/pycharm/

[19] Puledda F, Schankin C, Digre K, Goadsby PJ (2018) Visual snow syndrome: what we know so Far. Curr Opin Neurol 31:52–58. 10.1097/WCO.0000000000000523 29140814 10.1097/WCO.0000000000000523

[20] Yoo YJ, Yang HK, Choi JY, Kim JS, Hwang JM (2020) Neuro-ophthalmologic Findings in Visual Snow Syndrome. J Clin Neurol 16:646–652. 10.3988/JCN.2020.16.4.646 10.3988/jcn.2020.16.4.646 PMC7541978 33029971

[21] Lauschke JL, Plant GT, Fraser CL (2016) Visual snow: A thalamocortical dysrhythmia of the visual pathway? J Clin Neurosci 28:123–127. 10.1016/j.jocn.2015.12.001 26791474 10.1016/j.jocn.2015.12.001

[22] Eren O, Rauschel V, Ruscheweyh R et al (2018) Evidence of dysfunction in the visual association cortex in visual snow syndrome. Ann Neurol 84:946–949. 10.1002/ana.25372 30383334 10.1002/ana.25372

[23] Puledda F, O’Daly O, Schankin C et al (2021) Disrupted connectivity within visual, attentional and salience networks in the visual snow syndrome. Hum Brain Mapp 42:2032–2044. 10.1002/hbm.25343 33448525 10.1002/hbm.25343 PMC8046036

[24] Aldusary N, Traber GL, Freund P et al (2020) Abnormal connectivity and brain structure in patients with visual snow. Front Hum Neurosci 14:582031. 10.3389/fnhum.2020.582031 33328934 10.3389/fnhum.2020.582031 PMC7710971

[25] Vanneste S, De Ridder D (2012) The auditory and non-auditory brain areas involved in tinnitus. An emergent property of multiple parallel overlapping subnetworks. Front Syst Neurosci 6. 10.3389/fnsys.2012.00031 10.3389/fnsys.2012.00031 PMC3347475 22586375

[26] Puledda F, Schankin CJ, O’Daly O et al (2021) Original research: localised increase in regional cerebral perfusion in patients with visual snow syndrome: a pseudo-continuous arterial spin labelling study. J Neurol Neurosurg Psychiatry 92:918. 10.1136/jnnp-2020-325881 34261750 10.1136/jnnp-2020-325881 PMC8372400

[27] Schankin CJ, Maniyar FH, Chou DE et al (2020) Structural and functional footprint of visual snow syndrome. Brain 143:1106–1113. 10.1093/brain/awaa053 32211752 10.1093/brain/awaa053 PMC7534145

[28] Arnold W, Bartenstein P, Oestreicher E et al (1996) Focal metabolic activation in the predominant left auditory cortex in patients suffering from tinnitus: a PET study with [18F]deoxyglucose. ORL J Otorhinolaryngol Relat Spec 58:195–199 8883104 10.1159/000276835

[29] Stewart W, Wood C, Reed M et al (2008) Cumulative lifetime migraine incidence in women and men. Cephalalgia 28:1170–1178. 10.1111/j.1468-2982.2008.01666.x 18644028 10.1111/j.1468-2982.2008.01666.x

[30] Victor T, Hu X, Campbell J et al (2010) Migraine prevalence by age and sex in the united states: A life-span study. Cephalalgia 30:1065–1072. 10.1177/0333102409355601 20713557 10.1177/0333102409355601

[31] Puledda F, Schankin C, Goadsby PJ (2020) Visual snow syndrome. Neurology 94:e564–e574. 10.1212/WNL.0000000000008909 31941797 10.1212/WNL.0000000000008909 PMC7136068

