# Supplementary material for: Somatosensory perception in Visual Snow Syndrome: a comparison to age-, sex- and migraine-matched controls using Quantitative Sensory Testing - no evidence of altered somatosensory thresholds
Source: J Headache Pain. 2025 Nov 6;26(1):241. doi: 10.1186/s10194-025-02212-y (PMC12590906; doi:10.1186/s10194-025-02212-y)
Supplement: Supplementary file 1 — Supplementary Material 1 [file 10194_2025_2212_MOESM1_ESM.docx]

# Supplementary Material

| **Table 5**: Mean Z-score and standard error of mean (SEM) for QST parameters stratified by subgroup with respect to the control group with no migraine | | | | | | | | | | | |
| --- | --- | --- | --- | --- | --- | --- | --- | --- | --- | --- | --- |
|  | CDT | WDT | TSL | CPT | HPT | MDT | MPT | MPS | WUR | VDT | PPT |
| Patients with VSS and migraine | -0.479 ± 0.246 | -1.387 ± 0.698 | -0.153 ± 0.364 | 0.162 ± 0.309 | -0.541 ± 0.207 | 0.074 ± 0.289 | 0.380 ± 0.331 | -0.071 ± 0.394 | -0.040 ± 0.239 | -0.188 ± 0.381 | 0.191 ± 0.195 |
| Patients with VSS and no migraine | -0.425 ± 0.226 | -0.992 ± 0.527 | -0.121 ± 0.331 | 0.298 ± 0.271 | -0.404 ± 0.264 | -0.184 ± 0.281 | -0.068 ± 0.297 | -0.096 ± 0.272 | 0.189 ± 0.196 | -0.568 ± 0.350 | -0.308 ± 0.169 |
| Control patients with migraine | -0.021 ± 0.185 | -0.644 ± 0.501 | 0.127 ± 0.298 | 0.353 ± 0.270 | -0.428 ± 0.199 | 0.124 ± 0.203 | -0.431 ± 0.296 | -0.302 ± 0.375 | 0.369 ± 0.255 | -0.567 ± 0.199 | 0.802 ± 0.164 |

Z-scores were inverted for more clarity where necessary (namely for WDT, TSL, HPT, MDT, MPT and PPT) so that Z-scores above 0 indicate increased sensitivity compared to the control group (no VSS, no migraine)
